# Peripartum Cardiomyopathy: A Current Review

**DOI:** 10.1155/2010/149127

**Published:** 2010-07-18

**Authors:** Katie M. Twomley, Gretchen L. Wells

**Affiliations:** Section on Cardiology, Wake Forest University School of Medicine, Medical Center Boulevard, Winston-Salem, NC 27157-1045, USA

## Abstract

Peripartum cardiomyopathy (PPCM) is a rare but potentially lethal complication of pregnancy occurring in approximately 1 : 3,000 live births in the United States although some series report a much higher incidence. African-American women are particularly at risk. Diagnosis requires symptoms of heart failure in the last month of pregnancy or within five months of delivery in the absence of recognized cardiac disease prior to pregnancy as well as objective evidence of left ventricular systolic dysfunction. This paper provides an updated, comprehensive review of PPCM, including emerging insights into the etiology of this disorder as well as current treatment options.

## 1. Introduction

Peripartum cardiomyopathy, the development of systolic heart failure in the puerperium, is a rare but serious complication of pregnancy. Diagnosis requires objective evidence of left ventricular (LV) dysfunction with no other explanation for heart failure signs and symptoms [1, 2]. In the United States, the best estimate of incidence of peripartum cardiomyopathy (PPCM) is 1 : 3,000 live births [3]. While half of the women affected by this disorder recover LV function, the other half suffer significant morbidity and mortality [4]. The etiology of PPCM remains unclear, contributing to the poor outcomes seen in women affected by the disorder, as targeted treatment is not yet available.

## 2. Incidence

The incidence of PPCM in the United States is difficult to estimate as overlapping diagnosis codes make chart review both tedious and potentially inaccurate. Until recently, only small studies reporting the experience of single centers were available to estimate the incidence of this rate disorder. Two large studies in the United States reviewed ICD-9 codes and performed chart reviews to better report an estimate of incidence. Charts from discharges from the National Hospital Discharge Survey database (1990–2002) were reviewed to identify cases of PPCM. This study reported an estimated incidence of 1 : 3,189 live births in the US with the highest incidence occurring in African-American women [3]. A similar study examined ICD-9 codes within the database of the Kaiser Permanent health system in southern California from 1996–2005 and estimated an incidence of 1 : 4025 live births, again reporting the highest incidence in African-American women [5]. This study, however, had a high percentage of Hispanic women, the ethnicity with the lowest incidence of PPCM. Finally, a recent case-control study found an incidence of approximately 1 : 540 which was higher than that reported in other US series but comparable to that reported in African countries [6].

## 3. Risk Factors

The strongest risk factor for PPCM appears to be African-American ethnicity (OR 15.7; CI 3.5–70.6) [6]. Other reported risk factors include age, pregnancy-induced hypertension or preeclampsia [3], multiparity, multiple gestations, obesity, chronic hypertension, and the prolonged use of tocolytics [7].

## 4. Diagnosis

The National Heart, Lung and Blood Institute (NHLBI), with the National Institutes of Health (NIH), published diagnostic criteria for PPCM to direct more accurate research on epidemiology, pathophysiology, and outcomes. The criteria include: (1) onset of heart failure signs and symptoms in the last month of pregnancy or within 5 months postpartum; (2) LV systolic dysfunction with ejection fraction (EF) measured ≤45% or LV end diastolic dimension ≥2.7 cm/m^2^; (3) no evidence of pre-existing heart disease prior to peripartum symptom onset; (4) no other identifiable causes of heart failure [1]. Use of these criteria should prevent the inclusion of women with undiagnosed but pre-existing heart disease unmasked by the hemodynamic effects of pregnancy, as these women should present with signs and symptoms of heart failure in the second trimester when the hemodynamic stress of pregnancy peaks [8]. However, Elkayam et al. described women presenting with heart failure earlier in pregnancy with similar clinical courses and outcomes as women meeting the established diagnostic criteria [9]. An objective measurement of LV function excludes women with normal cardiac function with postpartum volume overload, which is common due to normal physiologic changes of pregnancy. Finally, PPCM is a diagnosis of exclusion [10] as many peripartum complications may result in depressed cardiac function, including infection, pulmonary embolism, and myocardial ischemia.

## 5. Clinical Findings

The clinical presentation of PPCM is most often dyspnea (90%), tachycardia (62%), and edema (60%) [11]. Some case studies also cite unusual presentations, including multiple thromboembolic events [12] and acute hypoxia [13]. Onset occurs one month prior to delivery and up to five months after delivery. However, the majority of women present postpartum. The most common clinical presentation (dyspnea, tachycardia, and edema) can be mistaken for another disorder, such as pneumonia or depression. Therefore, when a woman presents in the puerperium with these findings, an echocardiogram should be considered. 

Cardiac biomarkers, including B-type natriuretic peptide (BNP), are elevated in patients presenting with PPCM although these markers are not unique to PPCM. Elevations of troponin T (TnT) appear to have prognostic significance in this group. A TnT level ≥0.04 ng/mL at presentation predicts persistence of systolic dysfunction with a sensitivity of 55% and specificity of 91% [14]. Inflammatory cytokines (IL-2, TNF*α* and IL-6) are elevated in women with PPCM compared to pregnancy controls [15, 16]. However, these cytokines are elevated in patients with other cardiomyopathies.

ECG abnormalities are often noted on presentation, most commonly sinus tachycardia, nonspecific ST-T segment changes, LV hypertrophy, premature ventricular contractions, and bundle branch block [17]. However, these changes may be present in a number of disorders as well as normal states. Moreover, the ECG may demonstrate no significant changes [18].

Evaluation of suspected PPCM should include an echocardiogram. Average EF at diagnosis is often reported in the range of 20–30%; however, some women present with severe systolic dysfunction and an EF <20% [19]. Several studies have noted a worse prognosis in patients presenting with an EF <20% or LV end diastolic dimension >6 cm [4, 11]. LV thrombus is present at diagnosis in 17% of patients and conveys a poor prognosis [19].

Invasive evaluation, such as cardiac catheterization or endomyocardial biopsy, is often unnecessary for diagnosis or treatment. The pathology identified on endomyocardial biopsy is often nonspecific edema, inflammation, hypertrophy, and fibrosis [8]. Inflammation consistent with myocarditis is present in up to 50% of specimens [20, 21]. None of these findings are specific for PPCM.

## 6. Prognosis

Recovery of systolic function occurs in roughly half of affected women and usually occurs within 6 months of symptom onset [22]. A rapid recovery of EF is often seen in patients after initial diagnosis and diuresis [23]. EF > 45% at 2 months after diagnosis predicts full functional recovery in 75% of women with this result [19]. However, one recent study has reported mortality up to two years after diagnosis despite functional recovery [24]. Of the approximately 50% of women without full recovery of systolic function, many benefit from improved EF or functional status, while others have persistent or progressive systolic dysfunction leading to transplant or death [1].

## 7. Potential Etiologies

A proposed, but unlikely, etiology is nutritional deficiency. Selenium deficiency has been investigated given its association with heart failure in the third world independent of pregnancy. Forty percent of women with PPCM had low levels of selenium, but no correlation was found between selenium levels and the severity of cardiomyopathy [25]. Another study found no difference in selenium levels in patients with PPCM versus control patients [26].

A genetic predisposition is plausible in PPCM; however, little data exist to confirm this theory. Familial clustering has been reported [16], and regional distribution is present in Africa. The genetic predisposition is likely at the cellular/molecular level, involving either altered cell signaling for mitochondrial mediated apoptosis, inflammatory modulator activation, or altered immune response during pregnancy leading to infection or autoimmunity. Each of these abnormalities being dependent upon pregnancy fits with the clinical picture of PPCM, a phenotypically normal woman who develops cardiomyopathy in the setting of pregnancy, but often recovers normal systolic function postpartum.

Hormonal abnormalities could contribute as women with PPCM have been found to have lower levels of progesterone, prolactin, and estrogen during pregnancy, all hormones with a vasodilatory response preventing hypertension in the face of intravascular volume expansion [16]. Whether this finding is causative or associative is unclear.

Alterations in the immune response are normal in pregnancy as immunosuppression is necessary to ensure the safety of the fetus during development. A potential maladaptive response to pregnancy which may account for the development of PPCM is an autoimmune response to the cardiomyocyte. Antibodies recognizing unique cardiac tissue proteins have been described in the sera of patients with PPCM. In this same study, the sera from patients with other cardiomyopathies did not recognize the same cardiac tissue proteins [16].

Infectious agents invading the cardiomyocyte resulting in myocarditis have been described in PPCM. Given the immunosuppressed state of pregnancy, it is logical that pregnant women are more susceptible to infection or viral reactivation. Endomyocardial biopsy specimens from some patients demonstrate inflammatory changes consistent with myocarditis, as well as the presence of viral DNA in some patients with PPCM [20, 21, 27]. The exact role of viral infection or reactivation in the development and clinical course of PPCM remains unclear, and no convincing evidence exists that myocarditis is the primary etiology of this disorder. More recently, Sliwa et al. have found no significant difference in outcome in patients with PPCM infected with HIV as opposed to those not infected with HIV [24].

The most exciting recent insight into the etiology of PPCM involves a mouse model. Female mice with a deletion of *stat3* develop PPCM. Deletion of *stat3* results in enhanced oxidative stress which is associated with the generation of a 16 kDa prolactin derivate. This protein appears to be a key factor in the development of PPCM, leading investigators to treat patients with bromocriptine with early promising results [18, 28–30].

## 8. Treatment

Standard heart failure treatment is recommended for PPCM until the EF recovers. Medications include angiotensin converting enzyme inhibitors (ACE inhibitors) or angiotensin receptor blockers (ARBs), beta blockers, and diuretics. However, caution must be exercised as ACE inhibitors and ARBS are contraindicated in pregnancy. In addition, patients are advised regarding nonpharmacologic approaches such as salt restriction and the avoidance of offending medications (e.g., nonsteroidal anti-inflammatory drugs). Select patients may eventually benefit from cardiac resynchronization therapy, internal defibrillators, or cardiac transplantation. If systolic function normalizes, patients can discontinue heart failure medications without subsequent decompensation [19].

An important therapeutic option to consider is anticoagulation. Pregnancy conveys a hypercoagulable state that extends approximately 3 months postpartum. During this time, patients with PPCM and depressed systolic function are at high risk for thrombus formation and thromboembolic events [9]. Warfarin anticoagulation should be considered for prevention of morbidity or mortality from thromboembolism [31].

No specific treatment has been identified to significantly alter the morbidity of PPCM. Small trials have reported benefits of pentoxifylline, intravenous immunoglobulin, and bromocriptine. Pentoxifylline decreased TNF*α* levels and increased EF in patients with PPCM [32] although this finding has not been reproduced. Intravenous immunoglobulin (IVIG) improved EF compared to standard treatment in one small retrospective study of 6 cases compared to 11 controls [33]. More recently, a 16 kDa prolactin derivate has been causally related in PPCM [28]. Case reports of recovery from PPCM with bromocriptine treatment have been described [18, 28–30]. Larger studies will need to be performed to validate this treatment.

Transplant requirements in small series are reported to be 6 to 10% [11, 34, 35]. Transplant requirement was historically poor in this group due to more frequent and severe episodes of rejection, but has improved with advances in the treatment of rejection and is no different from transplant survival in patients with idiopathic dilated cardiomyopathy [36]. In patients not receiving transplant, mortality is usually secondary to progressive systolic dysfunction or sudden cardiac death, but in up to one third of patients is due to fatal thromboembolic events [9].

In addition to prescribing standard heart failure treatment, appropriate management of the patient with PPCM involves counseling regarding subsequent pregnancies. Women with persistent LV dysfunction are advised not to pursue further pregnancies. How to advise women with recovered LV function is more challenging. Some studies report good outcomes [37], and others report a high rate of recurrence [11, 38, 39]. A useful tool to help predict those patients whose LV function has recovered could be stress echocardiography, reported in a small series [40]. The lack of contractile reserve on dobutamine stress echocardiography may predict recurrence of systolic dysfunction in subsequent pregnancies [40]; however, this finding has not been investigated in a large series of patients.

## 9. Summary

Although rare, when a woman is diagnosed with PPCM, only 50% will be expected to fully recover cardiac function. Obstetricians should suspect the diagnosis, particularly if the patient has risk factors, including African-American ethnicity. Evaluation should include an echocardiogram to assess the LV systolic function. Treatment includes ACE inhibitors or angiotensin receptor blockers, beta blockers, and diuretics. Consideration should be given to anticoagulation. A number of causes are being investigated, including nutritional, infectious, and genetic, which, hopefully, lead to more targeted treatments.
